# *Mycobacterium tuberculosis* transmission in Birmingham, UK, 2009–19: An observational study

**DOI:** 10.1016/j.lanepe.2022.100361

**Published:** 2022-03-24

**Authors:** Timothy M. Walker, Marc Choisy, Martin Dedicoat, Philip G. Drennan, David Wyllie, Fan Yang-Turner, Derrick W. Crook, Esther R. Robinson, A. Sarah Walker, E. Grace Smith, Timothy E.A. Peto

**Affiliations:** a Centre for Tropical Medicine and Global Health, Nuffield Department of Medicine, University of Oxford, UK; b Oxford University Clinical Research Unit, Ho Chi Minh City, Viet Nam; c University Hospitals Birmingham NHS Foundation Trust, Birmingham, UK; d Nuffield Department of Orthopaedics, Rheumatology and Musculoskeletal Sciences, UK; e Oxford University Hospitals NHS Foundation Trust, UK; f TB Unit and National Mycobacterial Reference Service, UK Health Security Agency, UK; g NIHR Oxford Biomedical Research Centre, University of Oxford, UK

**Keywords:** Mycobacterium tuberculosis, Epidemiology, Whole genome sequencing, Seasonality, Transmission

## Abstract

**Background:**

Over 10-years of whole-genome sequencing (WGS) of *Mycobacterium tuberculosis* in Birmingham presents an opportunity to explore epidemiological trends and risk factors for transmission in new detail.

**Methods:**

Between 1st January 2009 and 15th June 2019, we obtained the first WGS isolate from every patient resident in a postcode district covered by Birmingham's centralised tuberculosis service. Data on patients’ sex, country of birth, social risk-factors, anatomical locus of disease, and strain lineage were collected. Poisson harmonic regression was used to assess seasonal variation in case load and a mixed-effects multivariable Cox proportionate hazards model was used to assess risk factors for a future case arising in clusters defined by a 5 single nucleotide polymorphism (SNP) threshold, and by 12 SNPs in a sensitivity analysis.

**Findings:**

511/1653 (31%) patients were genomically clustered with another. A seasonal variation in diagnoses was observed, peaking in spring, but only among clustered cases. Risk-factors for a future clustered case included UK-birth (aHR=2·03 (95%CI 1·35–3·04), *p* < 0·001), infectious (pulmonary/laryngeal/miliary) tuberculosis (aHR=3·08 (95%CI 1·98-4·78), *p* < 0·001), and *M. tuberculosis* lineage 3 (aHR=1·91 (95%CI 1·03–3·56), *p* = 0·041) and 4 (aHR=2·27 (95%CI 1·21–4·26), *p* = 0·011), vs. lineage 1. Similar results pertained to 12 SNP clusters, for which social risk-factors were also significant (aHR 1·72 (95%CI 1·02–2·93), *p* = 0·044). There was marked heterogeneity in transmission patterns between postcode districts.

**Interpretation:**

There is seasonal variation in the diagnosis of genomically clustered, but not non-clustered, cases. Risk factors for clustering include UK-birth, infectious forms of tuberculosis, and infection with lineage 3 or 4.

**Funding:**

Wellcome Trust, MRC, UKHSA

**Research in context**

**Evidence before this study**

We searched PubMed for English language research articles up to 6th December 2021 using the search term ‘tuberculosis’, interchangeably with ‘seasonality’ and ‘transmission’. Seasonal variation in tuberculosis diagnoses have been observed in multiple settings. A previous analysis of clusters defined largely on circumstantial evidence reported that seasonality may be restricted to those clustered cases only. Whole genome sequencing (WGS) data provide unprecedented precision for identifying clusters of tuberculosis, and thereby for precisely mapping epidemiological trends. Previous studies using WGS for tuberculosis epidemiology have described outbreaks; assessed patient and strain-based risk factors for transmission; and trace the origins of local epidemics back to sources. Individual patient social risk factors have previously been associated with an increased risk of transmission, as has strain lineage 2. However, WGS data have not yet been used to understand the impact of policy interventions, explore seasonal trends, or to provide local public health teams with details on where, when, and after whom to expect further clustered cases in their area.

**Added value of this study**

We exploit over 10 years of data obtained from dense sampling and WGS of *M. tuberculosis* isolates in Birmingham to understand epidemiological patterns of relevance to local practice and beyond. We find unambiguous evidence for seasonality among clustered cases and not among non-clustered cases. We show that some policy interventions have not had their desired effect in Birmingham, with a levelling-off in the decline in non-clustered cases after 2015, even though there is evidence of impact elsewhere in England. We assess both patient and strain-based risk factors for local cluster growth, showing that UK birth and lineages 3 and 4 are all independently associated with cluster growth in our setting. We also identify where in the city clustering remains largely local and where it does not, providing detailed information on local transmission patterns.

**Implications of all the available evidence**

Seasonal variation in tuberculosis diagnoses is different for clustered and non-clustered cases. This raises questions of whether this is due to amplification of transmission in-doors in the winter and a predominantly short incubation period, or whether other factors are responsible. However, whilst this epidemiological pattern has wider significance, other patterns we observed are potentially more locally determined. WGS is an essential tool to investigate local patterns with implications for where, when, and after whom to expect further cases of tuberculosis. These data should become key considerations in decisions about the deployment of public health resources, and should be repeated locally in other settings.

Alt-text: Unlabelled box

## Introduction

Before the COVID-19 pandemic, England saw a steep decline in tuberculosis incidence over the preceding decade, with numbers falling from 8280 in 2011 to 4655 in 2018.^1^ This decline in incidence is seen in both UK and non-UK born populations.^1^^,^^2^ Some of this success has been attributed to structural changes to tuberculosis services, including a move to pre-entry screening for tuberculosis disease in new migrants from 2011; the implementation of the Collaborative TB Strategy 2015–20 and the establishment of TB control boards; and a focus on testing for tuberculosis infection in people aged 16–35 who arrived from a high-incidence country within 5 years.^2^, ^3^, ^4^

The precise impact of each intervention on the declining incidence remains unclear,^2^ and the task of eliminating tuberculosis is only likely to get harder as managing the residual case-load is inevitably more complex. As the competition for public health resources will remain fierce in the aftermath of the COVID-19 pandemic, there is an imperative to design and implement efficient and high-value interventions if the WHO's targets for ending the TB pandemic by 2035 are to be achieved in England, and indeed elsewhere.^5^

Whole-genome sequencing (WGS) data have long been promoted as a tool of potential value in monitoring trends and designing tuberculosis control measures. Birmingham is England's second largest city whose population grew from 1·05 million in 2009 to 1·14 million in 2019.^6^ Birmingham's population is younger and more ethnically diverse than the English average, with almost 25% of residents born overseas, of whom almost half have been UK resident for over 10 years.^7^ Neighbouring Solihull, which is less diverse, has a population that grew from 205,000 in 2009 to and 216,000 in 2019.^6^ Tuberculosis incidence in Birmingham peaked at over 40 cases per 100,000 population in 2009.^8^ WGS of all *M. tuberculosis* cultures has been undertaken in Birmingham and Solihull for over 10 years, initially as research and latterly part of routine service provision that expanded nationwide in 2018. Here we explore 10·5 years of *M. tuberculosis* WGS data and associated meta-data from patients diagnosed in Birmingham and Solihull to investigate how this can reveal trends in transmission and disease control, potentially aid contact investigations, help assess the impact of interventions past, and help inform the design of future control strategies.

## Methods

### Sample selection

The Birmingham and Solihull tuberculosis service provides tuberculosis care to the populations of Birmingham and Solihull, who constituted the study population. One isolate from each patient who was resident at diagnosis in a postcode district covered by the service between 1st January 2009 and 13th June 2019 was cultured and sequenced on Illumina platforms (see supplementary Table S1 for postcode districts). Starting in 2012, all retrospective cultures were retrieved from a frozen archive at the UK Health Security Agency (UKHSA) mycobacterial reference laboratory, Birmingham. These were cultured either in liquid media Mycobacteria Growth Indicator Tubes (BACTEC™ MGIT™ 960, Becton Dickinson) or on Löwenstein-Jensen media, and DNA extracted as previously described.^9^ From 2012 isolates were cultured and sequenced prospectively. From 2015, DNA was extracted directly from early positive MGIT cultures as previously described.^10^ Only samples identified as *M. tuberculosis sensu stricto* were included, and where postcode details were available to place the patient in the catchment area.

### Bioinformatics

Short reads were mapped to the H37Rv (Genbank accession number NC_000962.2) *M. tuberculosis* reference genome using Stampy version 1.0.17.^11^ Repetitive regions were masked along-side four genes with previously noted high levels of artefactual variation (*tuf, rrs, rrl, Rvnt38*).^9^^,^^12^ SAMtools mpileup version 0.1.18^13^ was used to call variants based on a minimum read depth of 5x and at least one read on each strand. 76 samples for which <88% of the reference genome was called A,C,G,T were excluded. A single nucleotide polymorphism (SNP) threshold was used to generate clusters after multi-FASTA alignment, as previously described,^9^ whereby any sequence within the defined SNP threshold of another in a cluster was considered part of the same cluster. Python software is available.^14^

### Epidemiology

Data on date of diagnosis, sex, social risk factors, anatomical focus of the disease, postcode district, UK birth or year of entry into the UK were obtained from the national tuberculosis surveillance system at UKHSA. Data on *M. tuberculosis* lineage were derived from lineage specific SNPs extracted from the genomic sequences.^15^

The Birmingham and Solihull tuberculosis service use a generous (sensitive) 12 SNP threshold to cluster isolates, meaning that any sequence within 12 SNPs of another was clustered with that isolate. They then assess possible transmission events using phylogenetic and epidemiological data to optimise specificity. As we did not have data on shared time, space or contacts here, we base the primary analysis on clusters defined by 5 SNPs as this distance is more suggestive of person-to-person transmission than 12 SNPs.^12^^,^^16^ However, we repeated all analyses using the 12 SNP cluster cut-off as a sensitivity analysis.

### Statistical analysis

We used a segmented Poisson harmonic regression to characterise the temporal dynamics of incidence for clustered and non-clustered patients. The harmonic part consisted of a linear combination of sine and cosine transformations of time to capture seasonal variation in the data. The segmented part allowed two prespecified breakpoints, 2011 and 2015, to capture potential effects of public health policy changes described in the introduction. The significance of the seasonality and the breakpoints were assessed by likelihood ratio tests comparing models with and without sine and cosine functions and with 1, 2 or 3 segments in the trends around 2011 and 2015. As Birmingham and Solihull's population increased over the study period, we considered a version of the Poisson model in which Office of National Statistics mid-year estimates of the population sizes were included as an offset. To assess the impact of other effect, such as border effects where cases at the beginning and end of the study might be wrongly classified as non-clustered, we used a generalized additive Poisson model (as implemented by the mgcv R package)^17^ with spline smoothers on the time variables to allow visual characterization of the temporal trend with greater flexibility than segmented harmonic Poisson regression allows. The number of knots were automatically found by cross-validation to optimize the bias-variance trade-off.

Univariable logistic regressions were used to characterise the temporal trends in the proportions of male patients, UK-born patients, clustered patients, and patients with social risk factors among the cases. Univariable linear regression was used to characterise the relationship between the total number of patients resident in a postcode district and the number of patients clustered within and between those districts.

We conducted a time-to-event analysis of patient and strain-based risk factors for a future genomically related case emerging (our unit of analysis). We first used Kaplan-Meier plots to show the cumulative risk of a further case based on the characteristics of each case seen. Time was counted from the previous case (*t* = 0) to the next case or censored at 13 June 2019 when the last two patients in the study were diagnosed. The risk factors we explored included the presence of infectious forms of tuberculosis (pulmonary, laryngeal or miliary); social risk factors (for which we pooled illicit drug use, alcohol dependence, history of homelessness or time in prison, to increase power); whether patients were born in the UK or overseas; and *M. tuberculosis* lineage.

We then used a multivariable mixed-effects Cox proportionate hazards model to quantify the independent effect of each of the above risk factors. As perfectly sequential transmission between patients was highly unlikely within clusters, and as the characteristics of the previous patient might not therefore best predict the emergence of the next, we used the mean of each risk factor in a cluster at the time each additional patient was diagnosed. We also controlled for cluster size and the mean number of males in the cluster at the time. Cluster identifier was included as a random effect. We used a likelihood ratio test to compare models with and without interaction terms, ultimately excluding all interaction terms as none were significant (*p* > 0·05).

Missing data on country of birth were assumed to be missing completely at random and corresponding observations were dropped from the relevant regression analyses. Missing data on social risk factors were assumed to be missing not at random as these data are more likely to have been entered routinely by nurses if the risk factors had been present. For the purpose of the relevant regression analyses these observations were handled as if no risk factor were present.

Analyses were performed in STATA 17 and R 4.1.0.^18^

### Ethics

All work was undertaken as part of UKHSA (then known as Public Health England) service evaluation under the authority of the Health and Social Care Act 2012. As such, no additional research ethics committee approval was required.

### Role of the funding source

The funders had no role in the study design, in the collection of data, its analysis or interpretation, the writing of the report or the decision to submit for publication.

### Data statement

All data are available in the supplementary tables, including European Nucleotide Archive accession numbers.

## Results

Between 01 January 2009 and 13 June 2019 there were 1653 patients in Birmingham and Solihull with culture confirmed tuberculosis and an *M. tuberculosis sensu stricto* whole-genome sequence available for analysis. Among the retrospective isolates (2009–12), 64 could not be sequenced as these were missing. 695 (42·0%) were female and 958 (58·0%) were male. 448 (27·1%) were UK born, 1195 (72·3%) were non-UK born, and data were missing for 10 (<1%). 354/448 (79·0%) UK born patients had pulmonary, laryngeal or miliary tuberculosis, of whom 96 (27·1%) had at least one social risk factor (alcohol dependency, illicit drug use, history of homelessness, or time in prison). This compared to 707/1195 (59·2%) non-UK born patients who had infectious tuberculosis, of whom 38 (5·4%) had at least one social risk factor. 34 (2·1%) patients had TB meningitis, of whom 1 had a social risk factor, and 105 (6·4%) had TB osteomyelitis or spondylitis, of whom 4 had a social risk factor. Lineages 1–4 were represented by 214, 86, 704 and 649 isolates, respectively. 247 (38·1%) patients with lineage 4 were UK born compared to 15–21% for the other three lineages. 511/1653 (30·9%) patients could be genomically clustered with at least one other case (supplementary Table S2 and S3).

### Temporal trends in case-load

**Figure 1:**
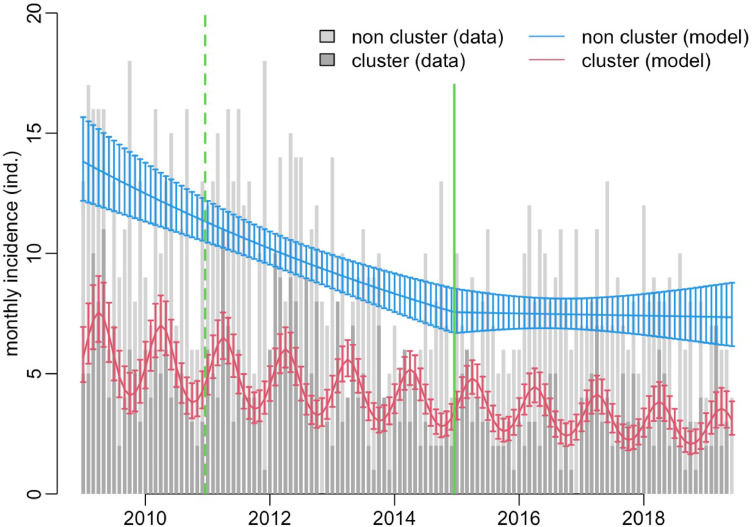
All cases from the beginning to end of the study. Red model shows seasonal trend in the diagnosis of patients in a 5 SNP cluster. Blue model shows trends for patients unrelated to another in the study. Vertical green lines indicate the introduction of the UK's new entrant screening programme (dashed) and the introduction of the national Collaborative TB Strategy (solid).

Cases declined at a rate of 6·7% (95% confidence interval 5·2–8·2) per year over the study period. Among these, clustered cases declined at a rate of 7·4% (4.7–10·0) per year whereas for non-clustered cases the trend changed from an 11% decline (7·2–14·7) per year prior to 2015 when the Collaborative TB Strategy was introduced, to a 3·7% (-3·7–10·6) decline subsequently (chi^2^=5·2, df=1, *p =* 0·023). Interestingly a seasonal trend was also seen, but only among clustered cases, with diagnoses of patients genomically linked to another case peaking in spring and corresponding troughs seen in the autumn (Figure 1). To check that this phenomenon was not an artefact of how we defined a cluster, we repeated the analysis using a 12 SNP threshold and found the same seasonal patterns as for the 5 SNP threshold, with a similar levelling off of the decline in non-clustered cases after 2015 (chi^2^=4·3, df=1, *p =* 0·038; supplementary Figure 1). Increases in Birmingham and Solihull's population were not responsible for these findings, nor were any effects around the start and end dates of the study that could have resulted in clustered cases being missed (supplementary Figure 2).

As cases steadily decreased over time, we found a significant increase in the proportion of patients who were male, and in the proportion of patients with a social risk factor (supplementary Figure 3).

### Transmission

To understand how future public health resources might best be invested we assessed risk factors for cluster growth associated with host, organism and location.

The number of new introductions into the catchment area was determined by defining a new strain as any clinical sample which was >5 SNPs from any other sample previously seen in the study; there were 1285 new introductions. Of 511 genomically clustered cases, 158 (30.9%) were clustered in pairs, 108 (21.1%) in triplets, and 245 (47·9%) were part of a clusters of 4 or more individuals (supplementary Table S4a; see S4b for 12 SNP clusters). 30/214 (14·0%) lineage 1 isolates were genomically clustered compared to 20/86 (23·3%) lineage 2, 202/704 (28·7%) lineage 3 and 260/649 (40·1%) lineage 4 (*p* < 0·001) (supplementary Figure 4).

**Figure 2:**
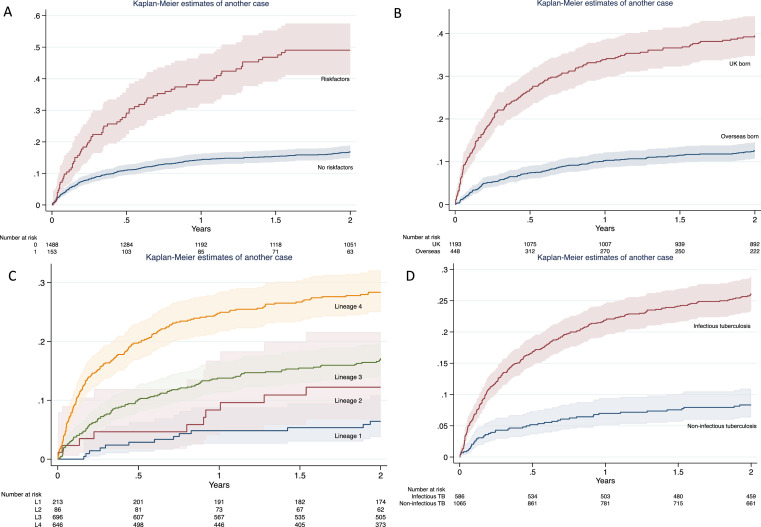
Risk of a future case arising in a cluster, given: A, the presence of one or more social risk factors B, where the patient was born; C, what lineage the patient is infected with; D, whether the patient has infectious (pulmonary, laryngeal or miliary) tuberculosis.

To understand how particular risk factors predict the emergence of another case in the cluster over time, we plotted Kaplan-Meier survival curves. In this univariable analysis, 38·6% (95% CI 30·8–46·8%) of patients with at least one social risk factor had already been followed by another case of tuberculosis in the same cluster after 1 year, rising to 47·1% (95% CI 38·9–55·3%) at two years. Similarly, 33·0% (95% CI 28·7–37·6%) of UK born patients were followed by another case within one year, and 37·9% (95% CI 33·4–42·6%) within 2 years. Among the different lineages, lineage 4 posed the greatest risk of another case at one year (24·2%, 95% CI 20·9–27·7) (Figure 2). Very similar results were seen for 12 SNP clusters (supplementary Figure 5).

A mixed-effects multi-variable Cox proportional hazards model assessed which risk factors, averaged over the cluster to date, were independently associated with a future case being diagnosed within the same cluster. Covariables included the presence of any social risk factors, UK vs. overseas birth, sex, *M. tuberculosis* lineage, the presence of an infectious form of tuberculosis (pulmonary, laryngeal or miliary), and cluster size. Cluster identifier was included as a random effect. Ten patients for whom their place of birth was unknown were excluded.

The mean number of patients in the cluster to date who had an infectious form of tuberculosis or who were UK born were the main patient centred factors associated with a future case emerging in a cluster (adjusted hazard ratio (aHR) 3·08, 95% confidence interval 1·98–4·78, *p <* 0.001; and aHR 2·03, 95% CI 1·36–3·04, *p =* 0·001, respectively). The cluster's *M. tuberculosis* lineage was also significant (aHR 1·91, 95% CI 1·20–3·56, *p =* 0·041 and aHR 2·27, 95% CI 1·21–4·26, *p =* 0·011 for lineages 3 and 4, respectively vs. lineage 1) (Table 1a). There was no evidence of differences by sex, cluster size, or the mean number of patients with a social risk factor. Very similar results were obtained after repeating this analysis with 12 SNP clusters, with the exception that mean number of social risk factors in the cluster to date was significant with the more relaxed threshold (Table 1b). See supplementary Table S5 for unadjusted hazard ratios, and supplementary Figure 6 for the mean interval days between cases by cluster size.

**Table 1:** Risk factors for a future, genomically related case being diagnosed.

	A future case is diagnosed				No future case is diagnosed					
	*N*	Risk factor present	Risk factor not present	% with risk factor	*N*	Risk factor present	Risk factor not present	% with risk factor	aHR (95% CI)	*p-*value
Risk of a future related case being diagnosed										
a. For clusters defined by 5 SNPs										
Pulmonary, laryngeal or miliary tuberculosis	367	309	58	84.2	1274	752	522	59.0	3·08 (1·98–4·78)	<0·001
Drug use	367	54	313	14.7	1274	39	1235	3.1		
Alcohol dependency	367	23	344	6.3	1274	16	1258	1.3		
Time spent in prison	367	38	329	10.4	1274	36	1238	2.8		
History of homelessness	367	20	347	5.4	1274	22	1252	1.7		
Composite 'any social risk factor' present	367	77	290	21.0	1274	76	1198	6.0	1·60 (0·87–2·93)	0·129
Born in the UK	367	189	178	51.5	1274	259	1015	20.3	2·03 (1·35–3·04)	0·001
Lineage 1	367	17	350	4.6	1274	196	1078	15.4		
Lineage 2	367	12	355	3.3	1274	74	1200	5.8	1·36 (0·52–3·53)	0·526
Lineage 3	367	139	228	37.9	1274	557	717	43.7	1·91 (1·03–3·56)	0·041
Lineage 4	367	199	168	54.2	1274	447	827	35.1	2·27 (1·21–4·26)	0·011
Male	367	227	140	61.9	1274	727	547	57.1	1·14 (0·78–1·66)	0·503
Cluster size after each consecutive case is diagnosed									1·00 (0·98–1·01)	0·641
										
b. For clusters defined by 12 SNPs										
Pulmonary, laryngeal or miliary tuberculosis	416	341	75	82.0	1225	720	505	58.8	2·36 (1·61–3·45)	<0·001
Drug use	416	61	355	14.7	1225	32	1193	2.6		
Alcohol dependency	416	25	391	6.0	1225	14	1211	1.1		
Time spent in prison	416	41	375	9.9	1225	33	1192	2.7		
History of homelessness	416	23	393	5.5	1225	19	1206	1.6		
Composite 'any social risk factor' present	416	87	329	20.9	1225	66	1159	5.4	1·72 (1·02–2·93)	0·044
Born in the UK	416	222	194	53.4	1225	226	999	18.4	3·16 (2·21–4·53)	<0·001
Lineage 1	416	20	396	4.8	1225	193	1032	15.8		
Lineage 2	416	13	403	3.1	1225	73	1152	6.0	1·28 (0·53–3·11)	0·581
Lineage 3	416	158	258	38.0	1225	538	687	43.9	2·12 (1·21–3·74)	0·009
Lineage 4	416	225	191	54.1	1225	421	804	34.4	2·56 (1·45–4·53)	0·001
Male	416	263	153	63.2	1225	691	534	56.4	1·35 (0·96–1·90)	0·084
Cluster size after each consecutive case is diagnosed									1·00 (0·98–1·01)	0·671

Results of mixed-effect Cox proportional hazards model shown as adjusted hazard ratio (aHR) for the mean number of patients with each risk factor in a cluster at the time each consecutive case in a cluster is diagnosed.

(1653 patients in the study, of whom 10 were excluded from this model as had missing data on place of birth, and 2 were censored as were diagnosed on the last day of the study, leaving 1641 patients in this Cox proportional hazards model).

**Figure 3:**
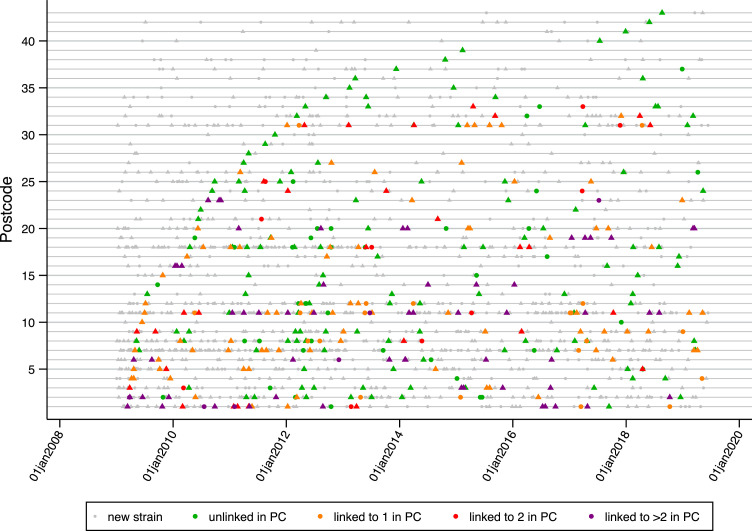
All patients in the study by date and postcode district where they lived at diagnosis. Dots indicate non-infectious TB, and triangles indicate infectious TB. Grey colour indicates patients whose strains are genomically unrelated to a previous strain and as such constitute new introductions. Green = related to one or more other strains, but not in the same postcode district. Orange = related to one other strain within the same postcode district, and possibly others in other postcode districts. Red = related to two other strains within the same postcode district, and possibly others in other postcode districts. Purple = related to more than two other strains within the same postcode district, and possibly others in other postcode districts. Postcode districts 1, 6 and 11 each have 2 clusters contributing 3 or more secondary cases. Where other postcode districts see 3 or more secondary cases, these are always from just one cluster.

We next explored in which 3-digit postcode districts transmission events based on 5 SNP clusters might be occurring. The median number of cases seen in a postcode district over the entire study period was 65 [inter-quartile range 30–127]. Postcode districts 7, 11 and 18 saw the most cases, with 141, 151 and 127, respectively, whilst postcode districts 39 saw just 2 cases in 10·5 years. We distinguished between patients with no prior genomically linked cases anywhere in the study, and those with a linked prior case. The latter might be considered preventable through contact tracing, whereas the former might not. Figure 3 shows all postcode districts, highlighting those that saw preventable cases.

**Figure 4:**
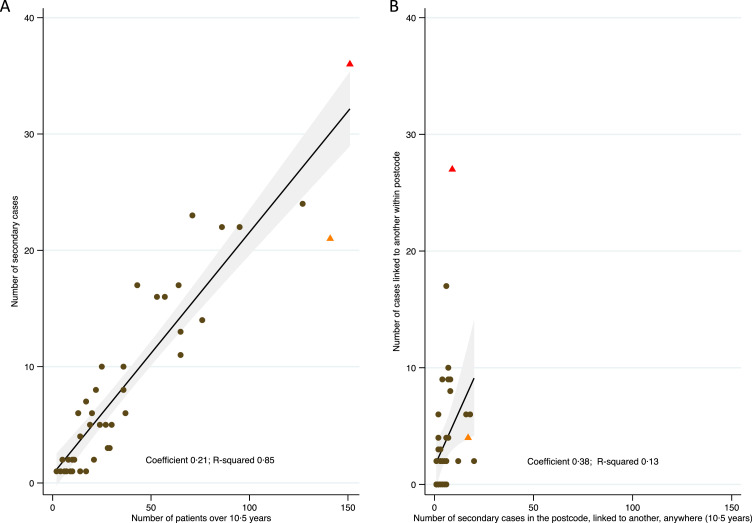
a: Number of cases over whole study period by the number of secondary cases in 5 SNP clusters. Data shown by postcode district. Linear regression line plots predicted mean with shaded area showing 95% confidence interval of the mean. 4b: Of patients who are genomically linked to at least one other one patient in Birmingham, the *y*-axis plots the number that are linked within the postcode district, and the *x*-axis the total number. The two postcode districts with the most number of cases overall are marked by red (postcode district 11) and orange (postcode district 7) triangles.

Linear regression was then used to characterise the relationship between the number of cases in a postcode district and the subset of secondary, or preventable, cases in that district from a source anywhere in the catchment area, over the study period. A mean of one additional secondary case for roughly every 5 cases in each postcode district was seen (coefficient 0·21, R^2^ 0·85, Figure 4a). However, among those cases with a related case somewhere in Birmingham or Solihull, a mean of one additional patient linked to another in the same postcode district was seen for roughly every 2·5 patients (coefficient 0·38, R^2^ 0·13, Figure 4b). There was significant heterogeneity across postcode districts, with postcode district 11 (having the highest number of cases) also seeing the highest proportion of within district clustering (75%), and postcode district 7 (having the second highest number of cases) seeing only low clustering (19%) (Figure 4a,b). Repeating this analysis for 12 SNP clusters produced very similar results (coefficient 0·22, R^2^ 0·84; coefficient 0·40, R^2^ 0·10, respectively, supplementary Figure 7a,b). Postcodes 7 and 11 are among the most deprived areas in Birmingham.

All data are available (see supplementary Table S2 for details).

## Discussion

Birmingham, England's second largest city, was the first in the world to start routine WGS of all *M. tuberculosis* samples for public health purposes. It was expected that the data would aid contact tracing and provide accurate data on trends in transmission and overall disease control.^9^^,^^19^ Here we present a largely prospective, 10-and-a-half-year population study of tuberculosis in the city. The findings demonstrate how routinely sequencing all culture positive samples can generate data to help direct when, around whom, and where to focus limited public health resources.

A steadily declining case-load was seen over the course of the study. Male sex and social risk factors became proportionally more common over time, independently of one another, and independently of country of birth. There was clear seasonal variation in diagnoses that applied to patients in clusters, but not to those who were unrelated to another case. Seasonality in tuberculosis diagnoses has previously been described, with speculation around the role of vitamin D deficiency in winter as a potential driver.^20^, ^21^, ^22^ Data from the USA also found that seasonality could be restricted to clustered cases, although those clusters were defined largely by spatio-temporal criteria.^23^ WGS data allow clusters to be defined with greater precision,^9^ and here for the first time help to unambiguously distinguish between two distinct epidemic trends; one seasonal, of recent transmission and early breakdown to disease, and another non-seasonal, of reactivation of disease after past infection and initial latency. Each requires different interventions. It could be that peaks in the spring and troughs in the autumn relate to indoor transmission in winter months.^24^ Clinical trial data suggests that Vitamin D deficiency is unlikely to be responsible.^25^ We are also unaware of any seasonal population changes in our setting. Our findings do however lend support to the case that the incubation period is often short, as the seasonal pattern is consistent with many patients developing tuberculosis within 6 months of infection.^26^

The Public Health England Collaborative TB Strategy and screening for tuberculosis infection in high-risk communities came into effect in 2015.^2^ It was therefore unexpected that the rate of decline among non-clustered cases levelled off after 2015. One could speculate that the impact of the introduction of pre-entrant screening in 2011 began to wear off by 2015, leaving a residual case load of UK-born patients, but there was only marginal evidence for an increase in the proportion of UK born patients over time (supplementary Figure 2). It is much less likely that the levelling off of the decline had anything to do with the new Collaborative TB Strategy, which was introduced to have the opposite effect.

We identified clear risk factors for the emergence of future cases within a genomically defined cluster, based on the characteristics of that cluster to date. Reflecting the finding of other population studies, a greater risk for an early additional case was seen in clusters enriched for UK-born patients and for patients with pulmonary, laryngeal or miliary tuberculosis.^16^^,^^19^ It is possible that UK-born patients are prone to later diagnosis, and therefore more transmission, if health providers don't consider tuberculosis within the differential in this population. Interestingly, infection with lineages 3 and 4 was also an independent risk factor, even after correcting for the random effect of cluster identifier. Social risk factors were only significant for 12 SNP clusters, perhaps as more geographically dispersed, and therefore less well sampled, clusters would have been better captured by the more generous threshold. Some of these clusters are known to the local tuberculosis team to be associated with social risk factors. Of particular relevance to public health teams tasked with outbreak control is the expected time to another case, based on individual patient risk factors. Although these were based on a univariable analysis (Figure 2), they are informative as predictors. These results show not just which characteristics of a patient, cluster or strain could be used to prioritise contact investigations, but also by when.

Others have reported that lineage 2 is more transmissible, including in the wider West Midlands region around Birmingham,^19^^,^^27^^,^^28^ or have linked transmissibility to non-lineage dependent genetic markers.^29^ A complex interplay between strain, host, and environment may explain some of the differences. However, we were able to study 511 patients in 143 clusters, sized 2 or more, and lineages 3 and 4 accounted for all six clusters with more than 10 patients (supplementary Table S2). The unusual density and duration of longitudinal population sampling in this study should lend credence to the findings, at least in this setting.

The finding that some postcode districts suffer from more local transmission than others suggests where efforts should be prioritised. Although local public health teams will be familiar with the higher-incidence postcode districts in the city, the findings that transmission in some high-incidence postcodes is predominantly local whereas transmission from others extends to different postcode districts should help better risk stratify investment in contact tracing.

There are limitations to this study. We did not have postcode data for all patients whose isolate was sequenced at the reference laboratory, so it is possible that we excluded some Birmingham or Solihull residents from the study. Additional samples were missing for the retrospectively collected cases, but will have been missing at random. No WGS data were available for culture negative samples, although culture negative patients are less likely to transmit the infection onwards. We will not have identified genomic links to patients outside of the temporal and geographic boundaries of the study, thereby missing some clustered cases. Moreover, contact tracing efforts throughout the study may have had a disproportionate impact on smaller, more simple clusters such as household clusters. WGS based contact tracing started in December 2016 and may also had an impact. It is possible that any of these factors could have introduced bias, but the finding of seasonality among clustered but not non-clustered patients is unlikely to have emerged if large amounts of patients were missing. Other limitations include missing data on social risk factors, especially in 2009, and missing data on HIV infection, diabetes, dialysis and patient age.

We did not explore direct person-to-person transmission as we lacked detailed epidemiological data. Approaches to reconstructing person-to-person transmission events exist,^30^ but we risk drawing false conclusions where data are missing. Our approach worked well for predicting future clustered cases based on the characteristics of the cluster to date, and should be of use to public health teams. Finally, we chose not to include isolates from after 2019 as the intention here was not to assess the impact of the COVID-19 pandemic on tuberculosis control.

We have nevertheless managed to densely sample Birmingham and Solihull for over a decade, using WGS and epidemiological data to generate a detailed picture of the public health challenge. WGS has now become routine in Birmingham and the rest of England. Although it has generated new work it has also saved resources previously spent on investigating links based on less specific molecular typing results. Our observation that seasonality is restricted to the diagnosis of clustered cases is likely to be more widely relevant. Practical interventions might include winter-time tuberculosis awareness campaigns for both healthcare workers and the community. Our approach to identifying which clusters are likely to grow, by when that might happen, and where those future cases might emerge may also provide useful to other settings. These are key pieces of information that can help guide the energies and resources of public health teams.

### Author contributions

TMW, DWC, TEAP, DW, MD, EGS, ER and ASW conceptualised the study. TMW, EGS, ER, DWC and DW contributed to data collected and curation. TMW, MC, ASW, PGD, FYT, and TEAP analysed the data. DWC, TEAP, ASW, EGS, MD and ER supervised the study. TMW wrote the first draft of the manuscript. All authors reviewed and edited the manuscript.

### Funding

The research was supported by the National Institute for Health Research (NIHR) Oxford Biomedical Research Centre (BRC). The views expressed are those of the author(s) and not necessarily those of the NHS, the NIHR or the Department of Health. We acknowledge the support of Wellcome Trust core funding (grant 098051) at the Wellcome Trust Sanger Institute. We acknowledge the support of the UKHSA (formally PHE). TMW is a Wellcome Trust Clinical Career Development Fellow (214560/Z/18/Z) but was an MRC research training fellow (MR/J011398/1) when this work started. DWC is an NIHR Senior Investigator. ASW is an NIHR Senior Investigator. EGS, ERR and DW are employed by UKHSA.

## Declaration of interests

No authors declare any conflict of interests.

## Appendix Supplementary materials

Image, application 1

Image, application 2

Image, application 2

Image, application 2

Image, application 2

Image, application 2

Image, application 2

Image, application 2
