# Supplementary figures and images for: Mycobacterium tuberculosis transmission in Birmingham, UK, 2009–19: An observational study
Source: Lancet Reg Health Eur. 2022 Mar 24;17:100361. doi: 10.1016/j.lanepe.2022.100361 (PMC8956939; doi:10.1016/j.lanepe.2022.100361)

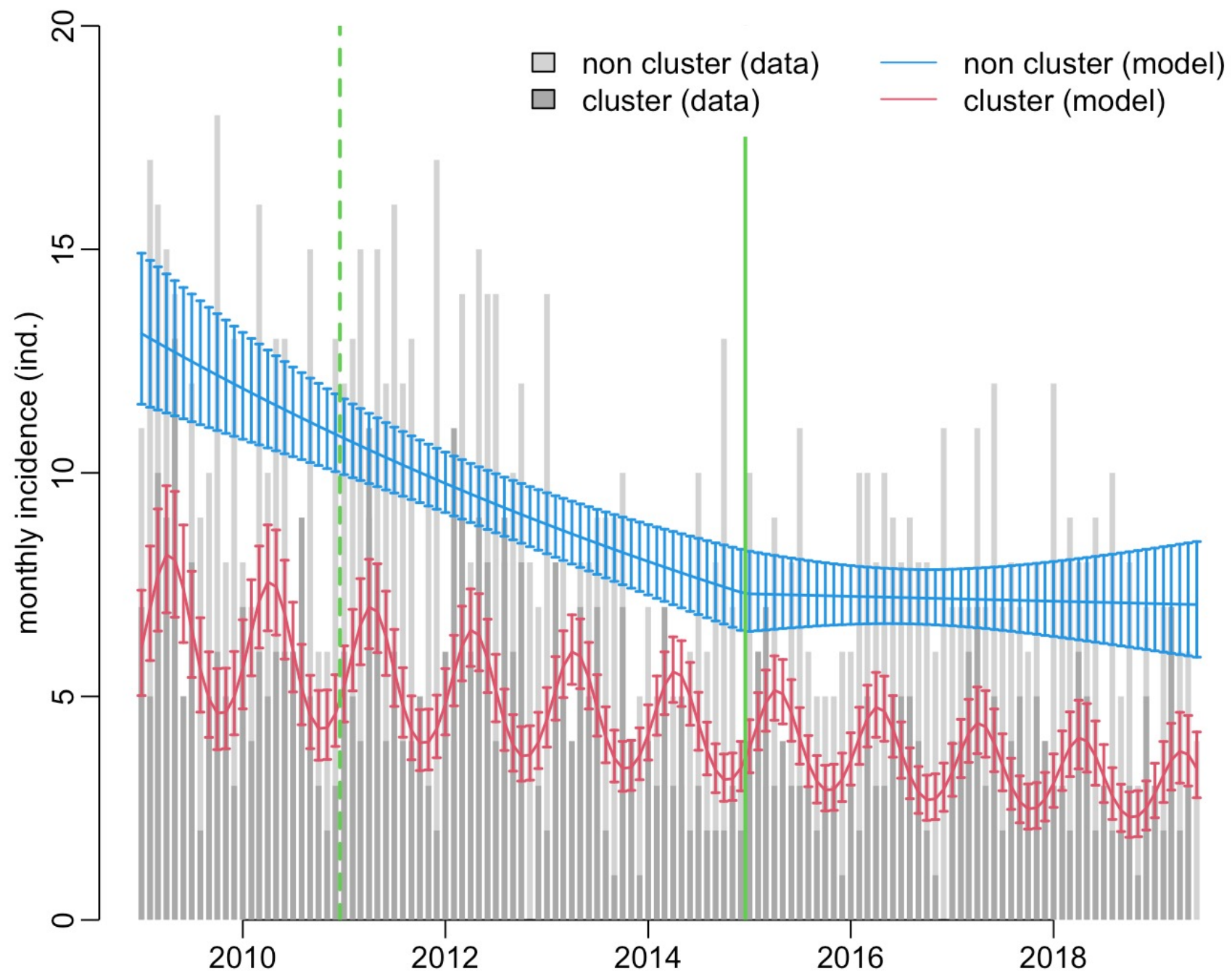

Supplement: Supplementary file 1 [file mmc1.pdf]

5 SNP

offset

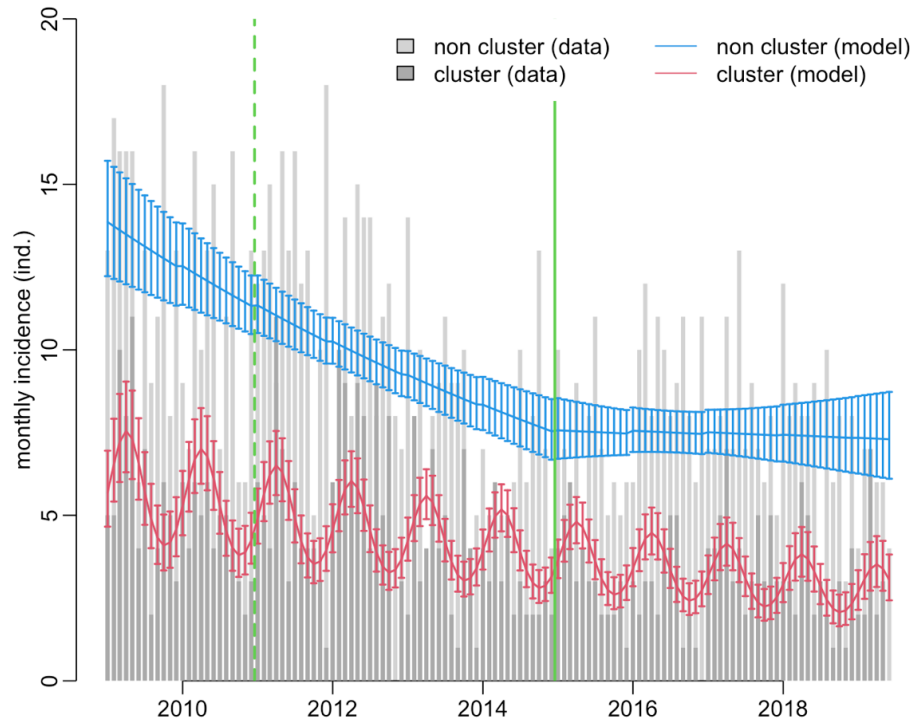

12 SNP

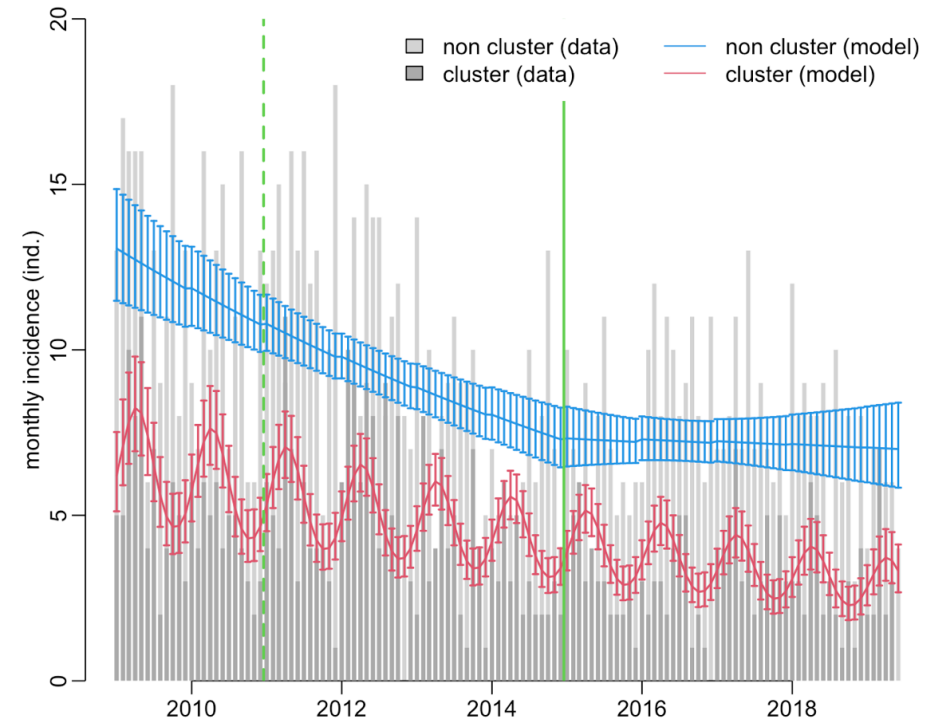

GAM

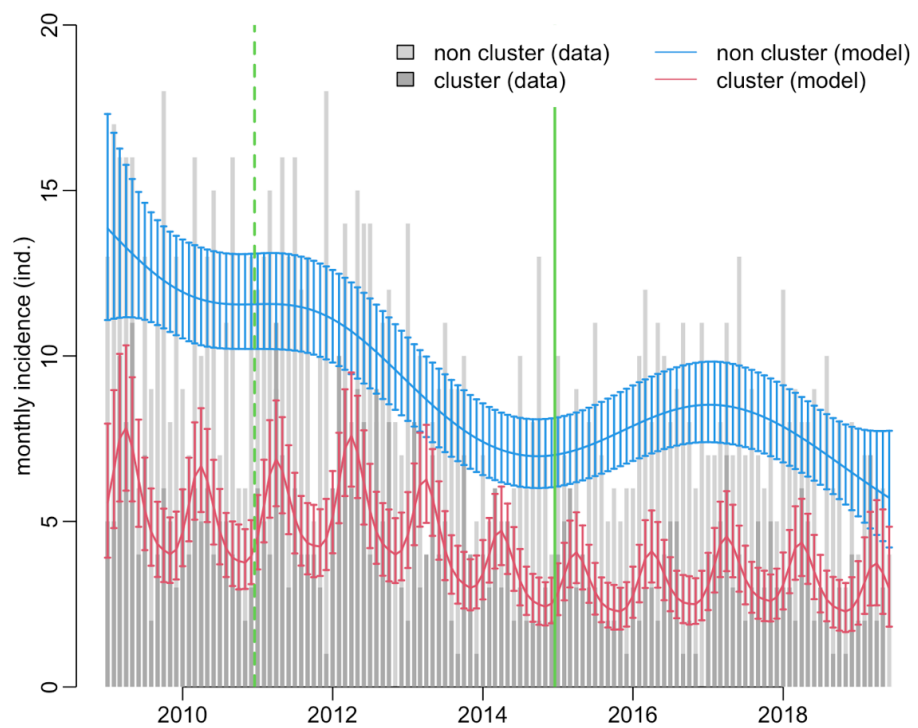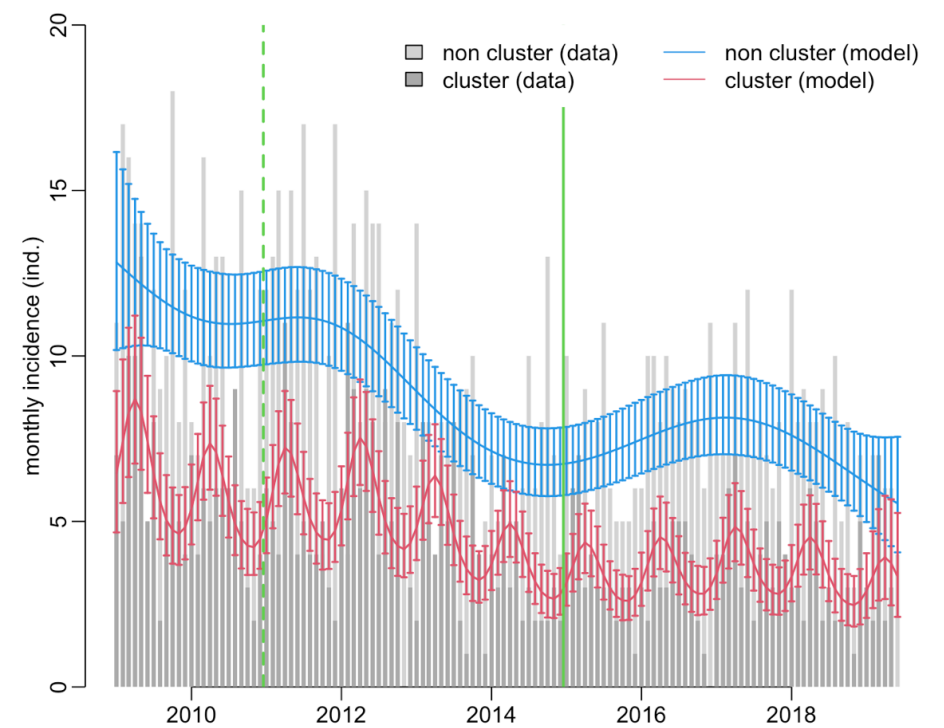

Supplement: Supplementary file 2 [file mmc2.pdf]

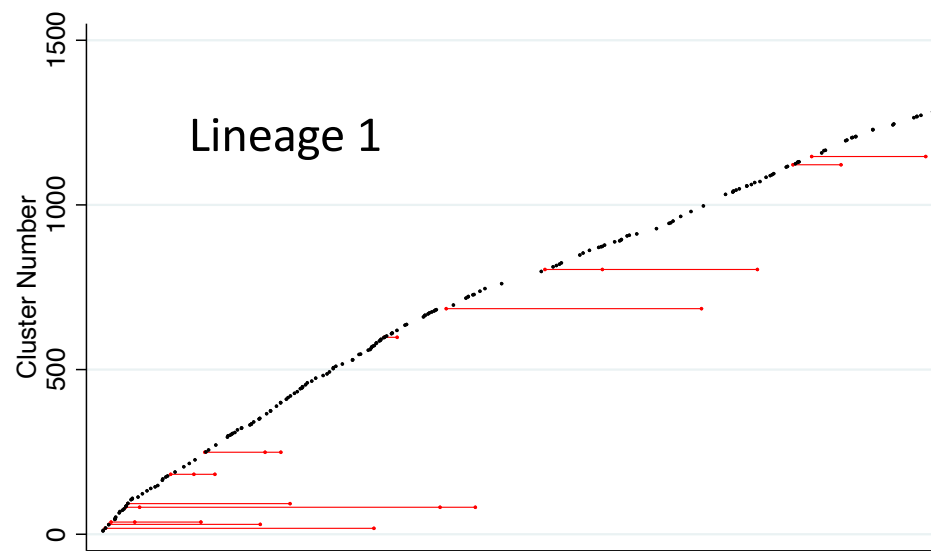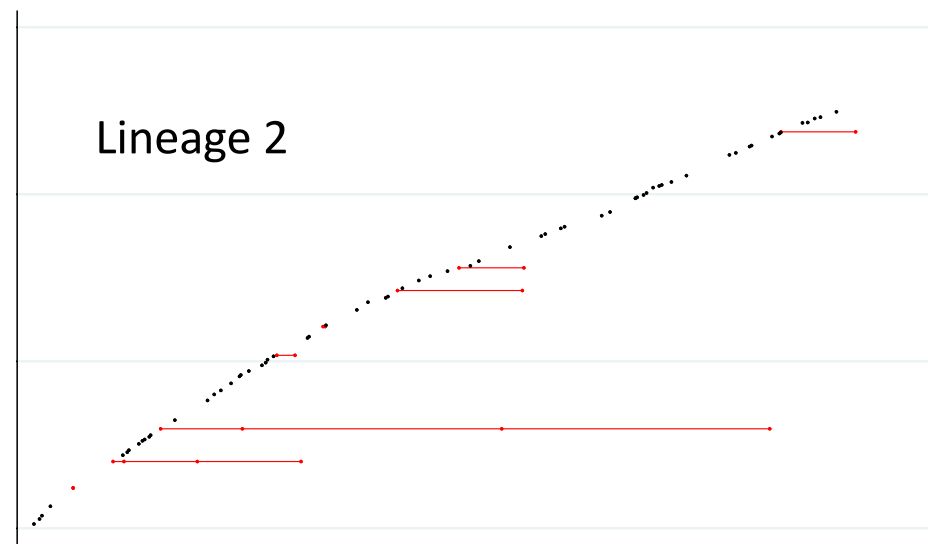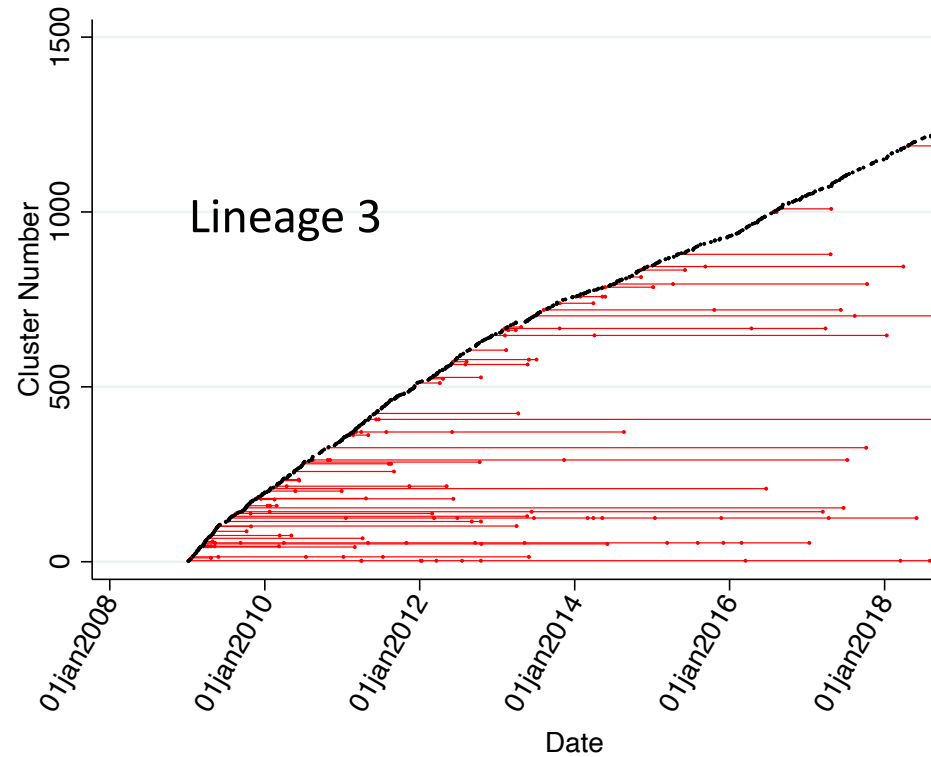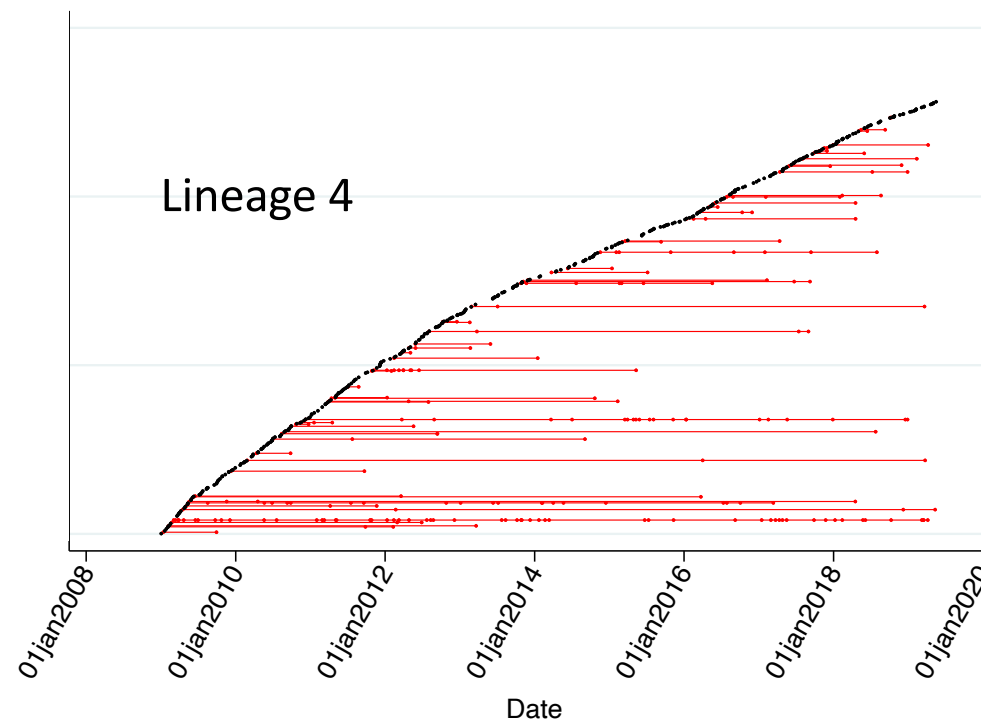

Supplement: Supplementary file 4 [file mmc4.pdf]

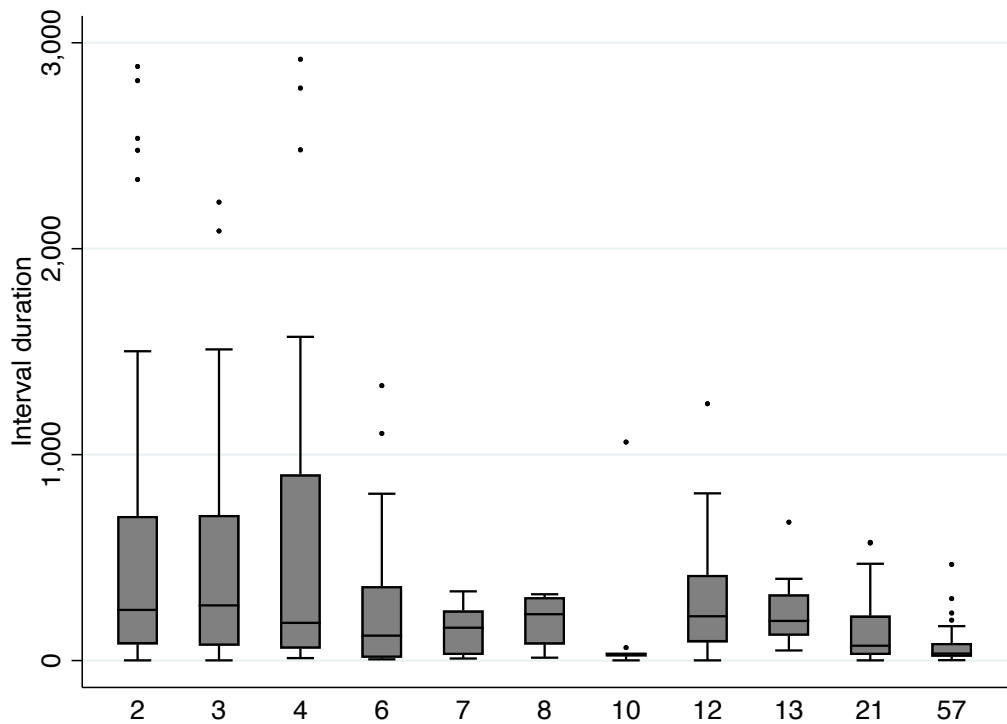

Supplement: Supplementary file 6 [file mmc6.pdf]

A

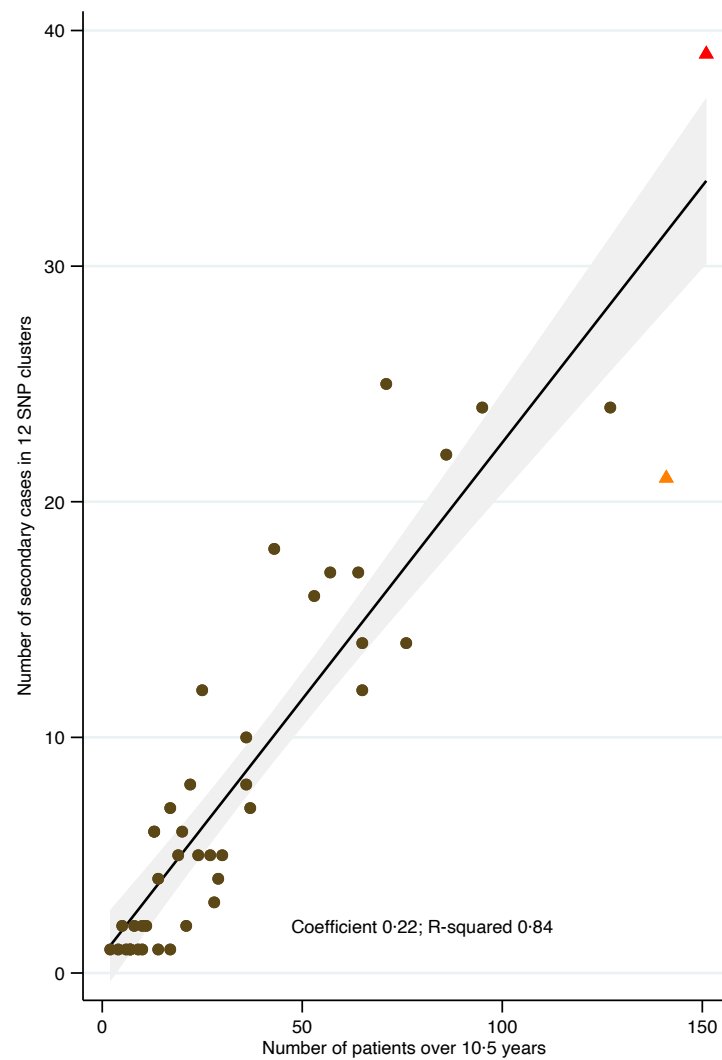

B

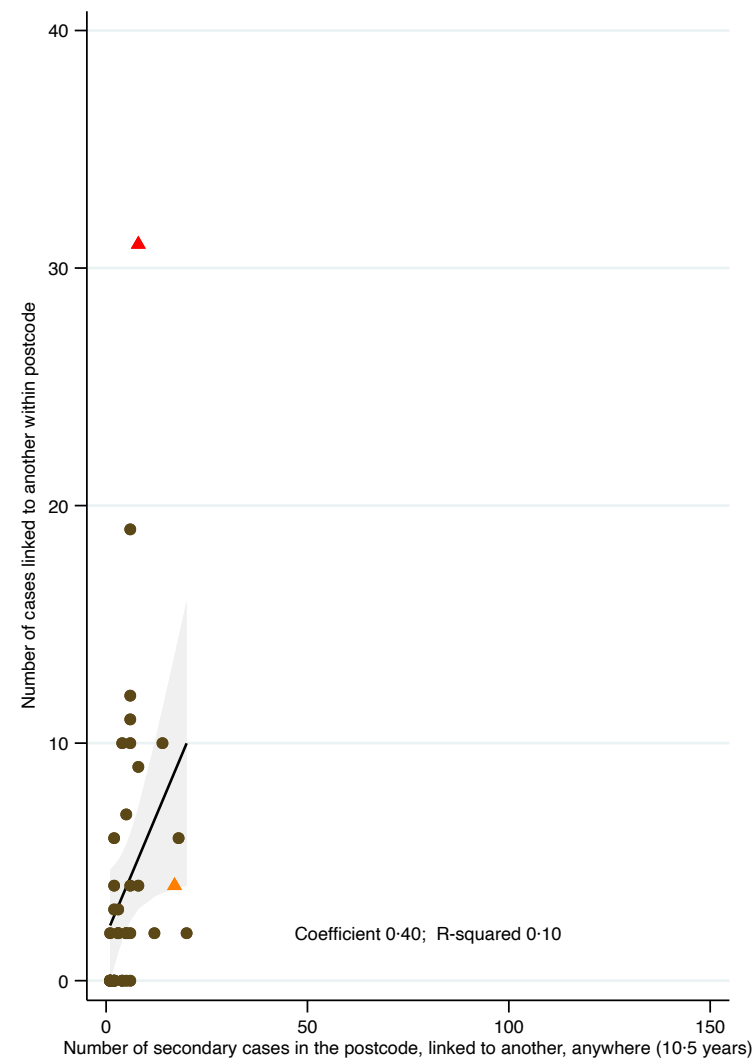

Supplement: Supplementary file 7 [file mmc7.pdf]
