# Supplementary material for: Mycobacterium tuberculosis transmission in Birmingham, UK, 2009–19: An observational study
Source: Lancet Reg Health Eur. 2022 Mar 24;17:100361. doi: 10.1016/j.lanepe.2022.100361 (PMC8956939; doi:10.1016/j.lanepe.2022.100361)
Supplement: Supplementary file 3 [file mmc3.pdf]

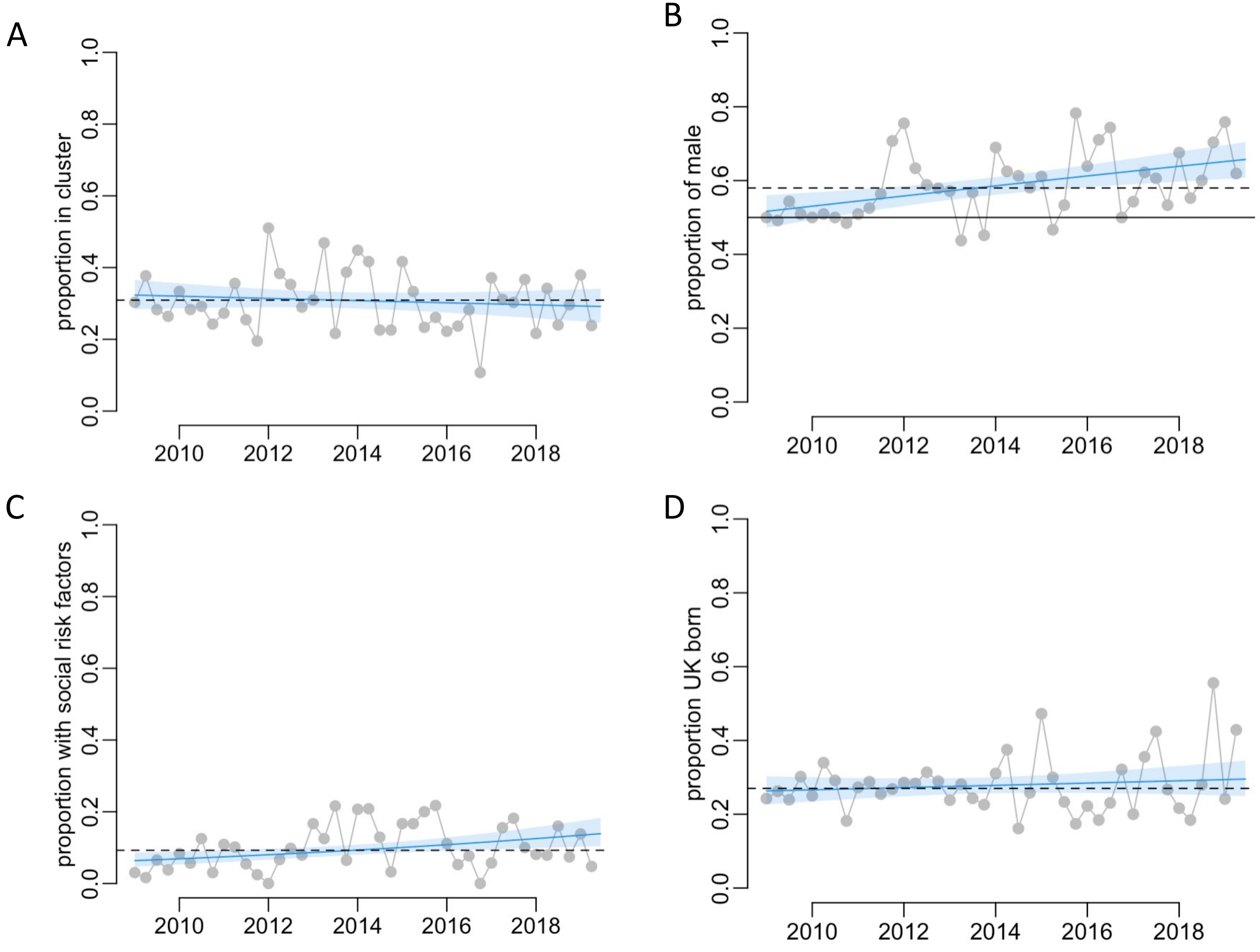

The significant increase in the proportion of male patients over time (panel B) is independent of the increase in the proportion of patients with social risk factors over time (panel C): p=0·003, based on logistic model assessing the significance of the temporal trend for panel B, after correcting for the temporal trend seen in panel C.
