# Supplementary material for: Mycobacterium tuberculosis transmission in Birmingham, UK, 2009–19: An observational study
Source: Lancet Reg Health Eur. 2022 Mar 24;17:100361. doi: 10.1016/j.lanepe.2022.100361 (PMC8956939; doi:10.1016/j.lanepe.2022.100361)
Supplement: Supplementary file 5 [file mmc5.pdf]

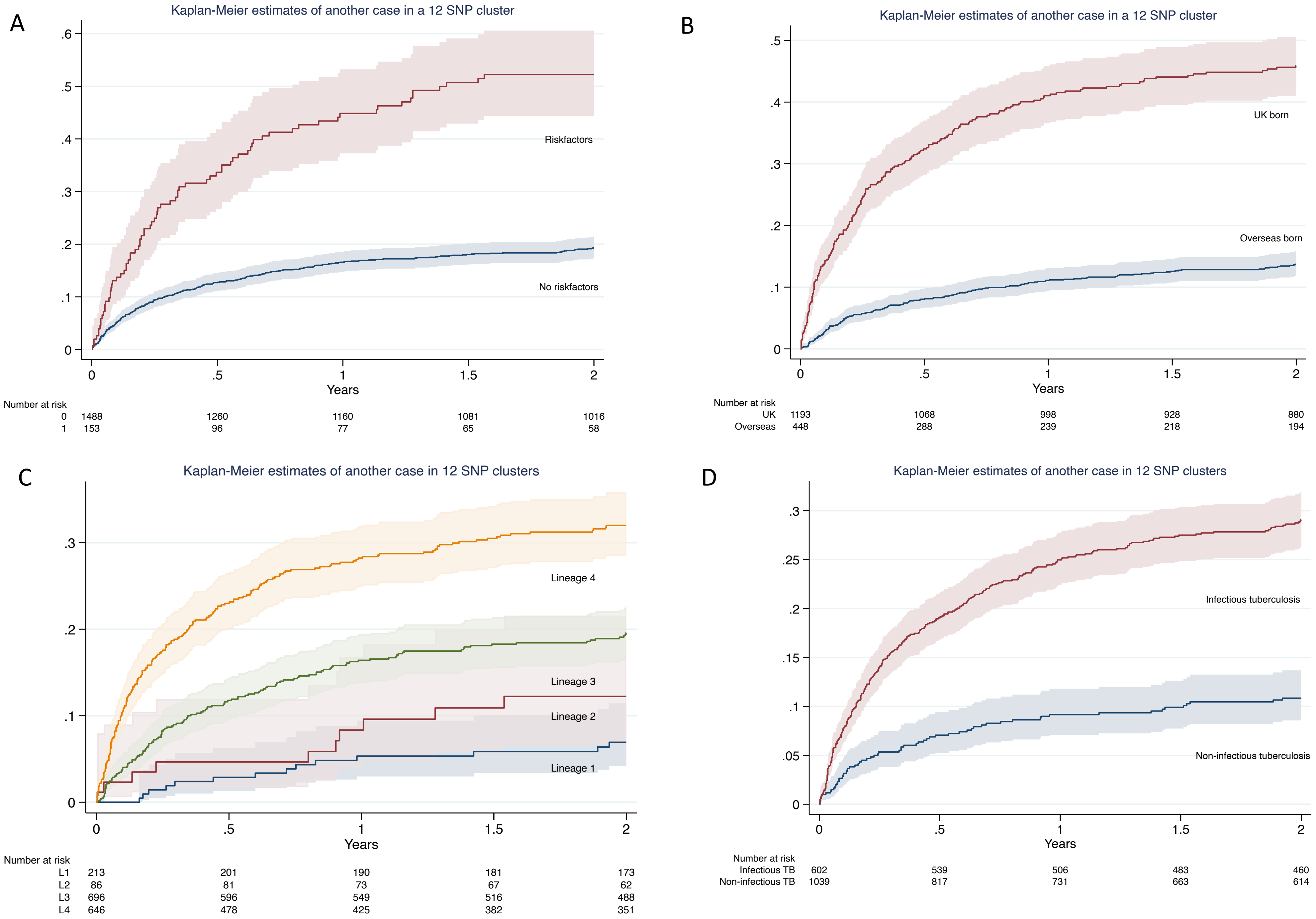

Supplementary Figure 4: Risk of a future case arising in a 12 SNP cluster, given: A, the number of social risk factors a patient has; B, where they were born; C, what lineage the patient is infected with; D, whether the patient has infectious (pulmonary or laryngeal) tuberculosis
